# The Diversity and Geographical Structure of *Orientia tsutsugamushi* Strains from Scrub Typhus Patients in Laos

**DOI:** 10.1371/journal.pntd.0004024

**Published:** 2015-08-28

**Authors:** Rattanaphone Phetsouvanh, Piengchan Sonthayanon, Sasithon Pukrittayakamee, Daniel H. Paris, Paul N. Newton, Edward J. Feil, Nicholas P. J. Day

**Affiliations:** 1 Lao-Oxford-Mahosot Hospital-Wellcome Trust Research Unit, Microbiology Laboratory, Mahosot Hospital, Vientiane, Laos PDR; 2 Department of Molecular Tropical Medicine and Genetics, Faculty of Tropical Medicine, Mahidol University, Bangkok, Thailand; 3 Mahidol-Oxford Tropical Medicine Research Unit, Faculty of Tropical Medicine, Mahidol University, Bangkok, Thailand; 4 Department of Clinical Tropical Medicine, Faculty of Tropical Medicine, Mahidol University, Bangkok, Thailand; 5 Centre for Tropical Medicine & Global Health, Nuffield Department of Clinical Medicine, University of Oxford, Oxford, United Kingdom; 6 Department of Biology and Biochemistry, University of Bath, Bath, United Kingdom; Beijing Institute of Microbiology and Epidemiology, CHINA

## Abstract

*Orientia tsutsugamushi* is the causative agent of scrub typhus, a disease transmitted by *Leptotrombidium* mites which is responsible for a severe and under-reported public health burden throughout Southeast Asia. Here we use multilocus sequence typing (MLST) to characterize 74 clinical isolates from three geographic locations in the Lao PDR (Laos), and compare them with isolates described from Udon Thani, northeast Thailand. The data confirm high levels of diversity and recombination within the natural *O*. *tsutsugamushi* population, and a rate of mixed infection of ~8%. We compared the relationships and geographical structuring of the strains and populations using allele based approaches (eBURST), phylogenetic approaches, and by calculating F-statistics (F_ST_). These analyses all point towards low levels of population differentiation between isolates from Vientiane and Udon Thani, cities which straddle the Mekong River which defines the Lao/Thai border, but with a very distinct population in Salavan, southern Laos. These data highlight how land use, as well as the movement of hosts and vectors, may impact on the epidemiology of zoonotic infections.

## Author Summary

Scrub typhus, caused by the pathogen *Orientia tsutsugamushi*, is endemic in Southeast Asia, including Laos, accounting for up to 15% of cases of undifferentiated fever in adult patients. Despite its public health importance, little is known about the genetics of the *O*. *tsutsugamushi* population in Laos—this information is important for optimizing diagnostics and epidemiological surveillance. We conducted a 4 year prospective study to examine the genetic diversity of *O*. *tsutsugamushi* causing scrub typhus in Lao patients and highlight the geographical differentiation that can occur even within a small country.

## Introduction

Scrub typhus, caused by the Gram negative obligate intracellular coccobacillus *Orientia tsutsugamushi*, is an important cause of acute febrile illness in Asia responsible for up to 23% of cases of undifferentiated fever [1]. The infection represents a major disease burden throughout a region ranging from northern Japan to Pakistan, to Russia in the north and northern Australia in the south. Over 55% of the world’s population lives in this densely populated endemic area [2]. It can affect patients of all ages, with at least one billion people living in rural areas at risk, and perhaps approximately a million patients needing medical attention every year [3]. Scrub typhus is transmitted to humans through the bite of infected larval trombiculid mites [4]. The clinical manifestations range from fever, headache, muscle pain, cough, and gastrointestinal symptoms, to coma, multi-organ failure and death [5].

In this study, we present data on the strain diversity and population structure of *O*. *tsutsugamushi* in Lao PDR (Laos), a country where the incidence of scrub typhus is almost certainly under-reported. Two recent studies found that up to 15% of adult patients with undifferentiated fever had scrub typhus [6, 7]. Molecular typing studies, aimed at understanding and monitoring the distribution of this disease, have been most commonly based on the highly polymorphic 56 kDa-outer membrane protein. This approach has indicated that the genotypes causing infection in Vientiane are similar to those circulating elsewhere in Laos and in Taiwan [8]. However, the use of a single surface protein gene marker can result in low resolution, or may provide misleading evidence concerning strain relatedness due to the confounding effects of recombination or immune selection. To address these shortcomings, we use multilocus sequence typing (MLST), which utilises sequences of multiple housekeeping genes. These are under less diversifying selection than surface protein genes, and are better able to both determine the relationships between closely related genotypes and reveal the genetic structure and mode of evolution of the bacterial population (clonal vs panmictic). Two alternative MLST schemes have been developed for *O*. *tsutsugamushi*; The first scheme characterised isolates from 84 Thai patients with scrub typhus, and revealed a highly diverse *O*. *tsutsugamushi* population with a very high rate of recombination [9]. Moreover, the rate of mixed infection, as indicated by ambiguous sequence at 1 or more loci, was as high as 25%. This high rate of mixed infection was not found by the authors who developed the second MLST scheme, although their scheme was applied to a relatively small number of cultured Cambodian strains rather than directly to patient blood [10]. Here we report the results of a 4 year prospective study aimed at determining the temporal dynamics and geographical structure of *O*. *tsutsugamushi* from patients from three different regions of Laos. We used the original MLST scheme (9) to characterise 74 isolates from these three different regions. The data confirm a highly diverse, recombining population, and reveal evidence for geographical structuring and local clonal expansion within Laos.

## Methods

### Patients and bacteria strains

**Fig 1:**
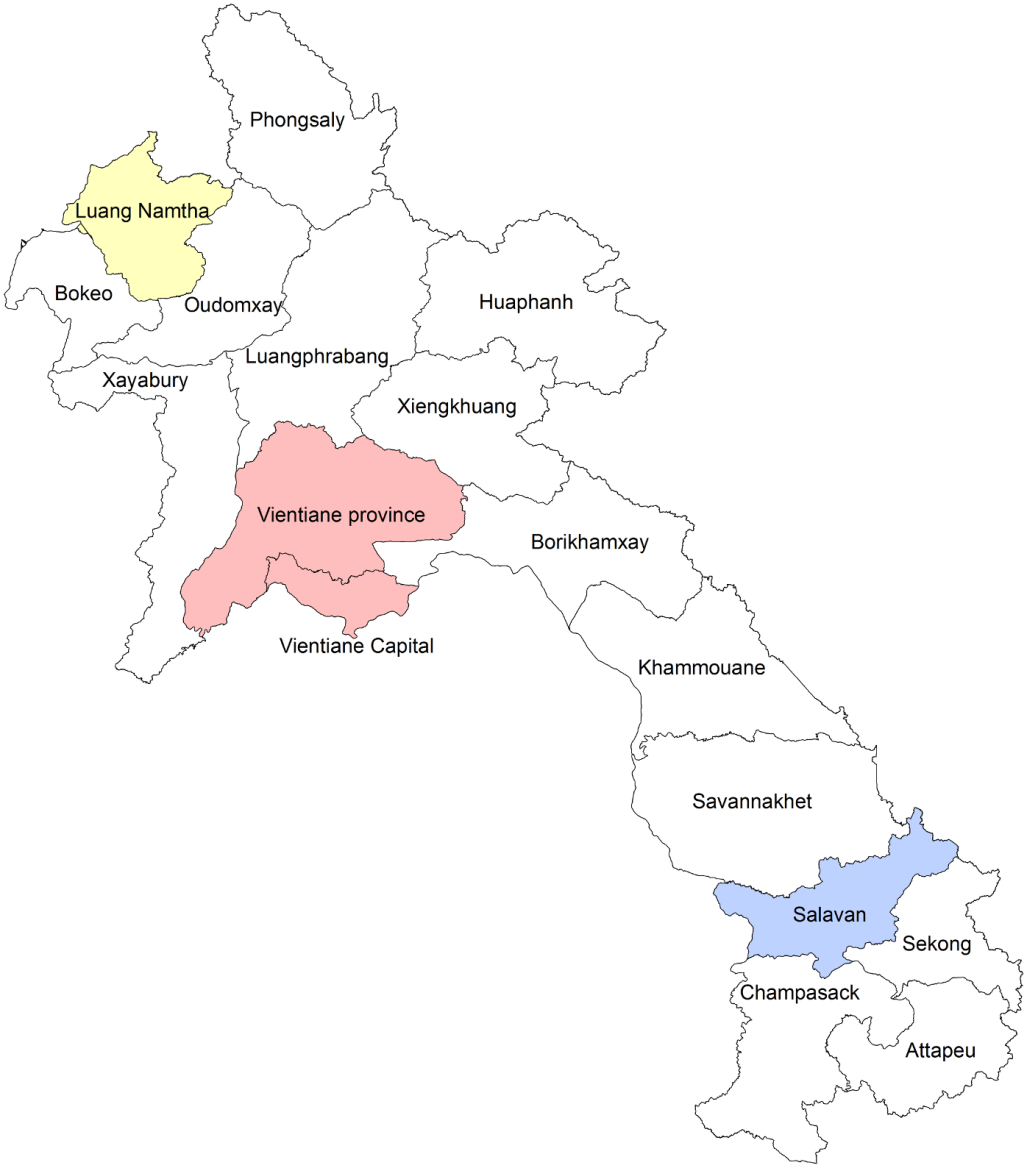
The geographic map of 3 study sites in Laos (Vientiane, Luang Namtha and Salavan).

A prospective study of patients presenting with acute fever to three hospitals in Laos from August 2008 to December 2012 was carried out. The hospitals, chosen to be in central, north and south Laos, were Mahosot Hospital in the capital Vientiane (17° 57ʹ N 102° 36ʹ E) [6], Luang Namtha Provincial Hospital in the northwest (21° 00ʹ N 101° 24ʹ E), and Salavan Provincial Hospital in the south (15° 43ʹ N 106° 25ʹ E) [7] (Fig 1). At Mahosot Hospital rickettsial blood culture was performed on all patients with suspected typhus. These were positive by point of care diagnostic test for either anti-*O*. *tsutsugamushi* IgM (CareStart assay; AccessBio, USA or Scrub Typhus IgM ICT, PanBio Inc., Australia) [11] or anti-*R*. *typhi* IgM (murine typhus Dip-Sticks IgM IBT, Panbio Inc., Australia) [12]. At Luang Namtha and Salavan patients with fever for ≤ 8 days, an admission tympanic temperature of ≥38°C, no obvious causes of fever (e.g. abscess, severe diarrhea, pneumonia), and negative malaria rapid diagnostic test had rickettsial culture performed.

### Ethics statement

This study was approved by the ethical review committee from the Lao National Ethics Committee for Health Research, Ministry of Health of Laos (No 25/NECHR), the Oxford Tropical Ethics Committee, UK and the Ethical Committee of Faculty of Tropical Medicine, Mahidol University, Thailand (Approval no. MUTM 2014-029-01). All patients subject in this study have provided written informed consent, and parents or legal guardians of any children participant provided written informed consent on their behalf.

### Bacterial isolation and identification

*O*. *tsutsugamushi* was isolated from EDTA blood by *in vitro* isolation as previously described [13]. Briefly, 5 ml of blood was drawn from the patient and centrifuged at 3,000 rpm for 10 min. 200 μl of the buffy coat was collected and mixed with 1ml of RPMI 1640 medium containing 10 mM HEPES (PAA, Austria) supplemented with 10% (v/v) fetal calf serum and transferred to L929 (mouse fibroblast) cell culture. The mixture was incubated in the presence of 5% CO_2_ at 35°C for 2 hours, the supernatant was removed and 5 ml new culture media were added and for further incubation. Cell culture media was changed three times per week by removing 2.5 ml media and replacing this with an equal volume of fresh media. Rickettsia infected samples were identified using indirect immunofluorescence assays as previously described [13]. Briefly, the bacterial culture was coated onto microscope glass slides and monoclonal antibodies for scrub typhus (STG-100), typhus group monoclonal antibody (TG-100) and spotted fever group monoclonal antibody (SFG-100) (Australian Rickettsial Reference Laboratory, Geelong, Australia) were added, followed by secondary goat anti-human IgA/M/G labelled with FITC (Invitrogen, USA). Fluorescence microscopy was used to identify cells infected with *Orientia /Rickettsia*. To confirm the presence of *O*. *tsutsugamushi* or *Rickettsia* spp., quantitative real-time PCR assays based on the 47 kDa outer membrane protein gene for identification of *O*. *tsutsugamushi*, 17 kDa surface protein gene for genus *Rickettsia* and *ompB* gene for *R*. *typhi* were performed as previously described [14–16].

### Multilocus sequence typing (MLST)

Genomic DNA of *O*. *tsutsugamushi* from the *in vitro* cell cultures was extracted using QIAamp DNA Mini kit 250 (QIAGEN, USA) and characterized using multilocus sequence typing (MLST) as previously described [9]. The housekeeping genes *gps*A, *mdh*, *nrdF*, *nuoF*, *ppdK*, *sucB*, *sucD* were amplified by PCR and sequenced in both directions using nested primer pairs (Table 1). The sequence data were edited and analyzed using SeqMan from LaserGene software (DNASTAR Inc., Wisconsin, USA) and allele numbers assigned by reference to previous data [9]. The MLST scheme for *O*. *tsutsugamushi* is hosted on the PubMLST website (http://pubmlst.org/otsutsugamushi/) [17]. The relationships between the STs were visualized using two implementations of the BURST algorithm [18]; e-BURST v. 3 and goeBURST [19]. The dN/dS of the 7 housekeeping gene partial sequences were calculated using START2-Sequence Type Analysis and Recombinational Tests, (http://pubmlst.org/software/analysis/start2/) [20]. A neighbour-joining tree of the isolates was constructed based on the 2,700 bp concatenated sequence of all loci (*gpsA-mdh-nrdF-nuoF-ppdK–sucB-sucD*) using MEGA version 5 [21]. The data in the current study was supplemented with existing data from Thailand [9]. The amount of genetic differentiation between populations from different geographical locations was estimated using F-statistics (F_ST_), which reflect the rates of migration, mutation and drift [22]. The estimation of the recombination per mutation ratio (r/m ratio) was calculated by comparing the sequences of non-identical alleles in all single locus MLST variants with their clonal founders. Multiple nucleotide changes (>1) were assumed to be caused by recombination while single nucleotide differences not found elsewhere in the database were assumed to be due to *de novo* mutation [23].

**Table 1:** List of 7 housekeeping gene and primer sequences used in this study.

Gene	Gene name	Primer	1^st^ PCR sequence (5'->3')	Productsize (bp)	Primer	2^nd^ PCR sequence (5'->3')	Sequence read size (bp)
*gpsA*	glycerol-3-phosphate dehydrogenase	gpsA_F	TCAGCCCATACTCAAGAAATCA	572	gpsA_NF	TCAGCTGCATACTAATAAAAA	390
		gpsA_R	GCAAATGCCACAATTTCCTT		gpsA_NR	GATGCTTTACAGTTTTGACCA	
*mdh*	malate dehydrogenase	mdh_F	CCAAAGCAGTTGCTCAAGGT	608	mdh_NF	AAAGCATGGGTATTGGTAAA	348
		mdh_R	AGCTGCTGCTGGAGCATAAT		mdh_NR	TCCTCCATCTCTAGTTCTTTGT	
*nrdF*	ribonucleoside-diphosphate reductase beta subunit	nrdF_F	TAAAGCATGGCACACTCAGC	595	nrdF_NF	AAATTCACTGGCTACCAGAA	384
		nrdF_R	CTGTTCTGTCCAAACTTCAGGA		nrdF_NR	TGTTTCATCTCTAACTGACCA	
*nuoF*	NADH dehydrogenase chain F	nuoF_F	ATCTGGTTCTATGGCAGTTGAC	645	nuoF_NF	AAAATCTGGCTTACGTGGT	360
		nuoF_R	CATTTTGCGCCTCTTCTGAGTA		nuoF_NR	GAGTATTGTCGGAACTACAGC	
*ppdK*	pyruvate, phospate dikinase precursor	ppdK_F	CAAAGGTGTAACACTTGCTCAGA	591	ppdK_NF	TACCTATACCGCATGGTTTT	396
		ppdK_R	TGGTGGTTCATCCATGATTTT		ppdK_NR	ACTGCTTGAATAGCTTGGTG	
*sucB*	dihydrolipoamide S-succinyltransferase	sucB_F	CAGCAAAAGAAAGATGTTCAGC	590	sucB_NF	ATTGGCACAACTAATCCAGA	411
		sucB_R	GGTTGCCAAAATGGTAGCAG		sucB_NR	GCATAAAATCAATCCTGAGAA	
*sucD*	succinyl-CoA synthase alpha chain	sucD_F	ATGTTCCTCCAGCTTTTGCT	599	sucD_NF	TGAAGCTATTGATGCTGGTA	411
		sucD_R	TCCAGCGCTTTTTAATGCTT		sucD_NR	AGCGCTTTTTAATGCTTCTA	

## Results

### Bacterial isolation and diversity

A total of 2,844 patients presenting with acute fevers were recruited from the three hospitals: 1,401 from Luang Namtha, 893 from Mahosot hospital and 550 from Salavan. A total of 195 (6.8%) of these patients were culture positive for *O*. *tsutsugamushi*, 58 from Luang Namtha (4.1% of all patients from this hospital), 118 from Vientiane (13.2% of all patients from this hospital), and 19 from Salavan (3.4% of all patients from this hospital). The relatively high percentage of patients from Vientiane confirmed as scrub typhus positive may reflect difficulties incurred during the transportation of samples from the other two areas. A total of 215 isolates were confirmed by both IFA and PCR, of which 195 (90.7%) were scrub typhus and 20 (9.3%) were *R*. *typhi* (Table 2). The first 81 out of 195 (41.5%) *O*. *tsutsugamushi* isolates were selected for MLST; 74 (91.3%) were successfully amplified and sequenced, while the remaining 7 isolates produced poor quality data, most likely as a result of mixed infection. Of the final 74 isolates, 51 originated from Vientiane, 11 from Luang Namtha and 12 from Salavan. These 74 isolates corresponded to 50 different sequence types (STs), 43 of which were novel to this study (STs 50 through ST 92). Simpson’s index of diversity was calculated as 0.98 (95% CI 0.97–0.99) confirming a highly diverse population [24]. The seven STs that were not novel had been previously reported by Sonthayanon et al. in a study of *O*. *tsutsugamushi* from patients presenting to a hospital in Udon Thani, Northeast Thailand [9]. In the current study, the isolates corresponding to these seven previously recorded STs all originated in Vientiane, which is on the Laos/Thai border. These shared STs largely correspond to the clonal complexes previously described in the Thai study; ST29 and ST30 correspond to CC29, ST37 and ST25 correspond to CC37, ST9 corresponds to CC10 and ST4 corresponds to CC13. The remaining ST common to both studies was ST1 which is a single locus variant (SLV) of ST2, the second most common ST noted in the Thai study [9]. This is consistent with the view that these clusters are commonly encountered in both Thailand and Vientiane, although they appear not to have made significant incursions to other regions of Laos.

**Table 2:** *In vitro* isolations from patients recruited in Laos during August 2008 to December 2012.

Location (Province)	Number of samples (%)	Culture positive by direct immunofluorescence method	
		Scrub typhus group (%)	Typhus group (%)
Mahosot (Capital)	893 (31.3)	118/893 (13.2)	20/893 (2.2)*
Luang Namtha (North)	1401 (49.3)	58/1401 (4.1)	0
Salavan (South)	550 (19.4)	19/550 (3.4)	0

*All of these were *Rickettsia typhi*

Of the novel STs, 34 originated from Vientiane, 7 from North-Laos (Luang Namtha) and 9 from South-Laos (Salavan). The proportions of novel STs are therefore very similar in Vientiane (66%) and Luang Namtha (63%), but slightly higher in Salavan (75%). These novel STs did not simply reflect different combinations of previously described alleles, as might be expected in this highly recombining species, but also new allele sequences. 18 new alleles were noted for *gpsA*, 6 for *mdh*, 13 for *nrdF*, 16 for *nuoF*, 17 for *ppdK*, 8 for *sucB* and 14 for *sucD*. This again points to considerable population diversity, both in terms of the overall number of STs, but also in terms of the number of alleles per locus.

The most common sequence type was ST86, which was represented by 7 isolates, all from Vientiane (Table 3). ST37, ST58, and ST71 were each represented by 3 isolates, and were also recovered from a single origin (the three ST37 isolates were all from Vientiane, the three ST58 isolates from Luang Namtha and the three ST71 isolates from Salavan). Twelve STs represented by two isolates each were noted, nine of which originated exclusively from Vientiane (STs 1, 4, 9, 30, 51, 59, 67, 78), with one pair from Luang Namtha (ST65) and one pair from Salavan (ST75). There was a single occurrence of an ST being recovered from more than one region; ST69 corresponded to one isolate from Luang Namtha, and one from Vientiane. In summary, whilst the majority of the STs (34/50; 68%) are only represented by a single isolate, in those cases where a single ST is represented by multiple isolates those isolates exhibiting the same ST also originate from the same region (with the exception of a single isolate pair). Given that a pair of isolates drawn at random from the data would be expected to originate from the same region only 51.6% of the time (calculated by summing the probabilities that a random pair of isolates both correspond to one of the three regions), this observation is therefore strongly indicative of geographical clustering and the local clonal expansion of specific STs.

**Table 3:** Sequence type of 74 *O*. *tsutsugamushi* isolates from Laos. The strain name prefix TM indicates samples from Vientiane, LNT those from Luang Namtha, and SV those from Salavan.

Strains name	ST	MLST loci						
		*gpsA*	*mdh*	*nrdF*	*nuoF*	*ppdK*	*sucB*	*sucD*
TM 2002	1	1	1	1	1	1	1	1
TM 2325	1	1	1	1	1	1	1	1
TM 2755	4	2	2	1	2	2	2	2
TM 2758	4	2	2	1	2	2	2	2
TM 2863	9	3	3	2	1	3	4	4
TM 2981	25	10	8	8	9	10	2	5
TM 2309	29	11	9	10	10	7	7	10
TM 2395	29	11	9	10	10	7	7	10
TM 1084	30	11	9	10	10	7	8	10
TM 2304	30	11	9	10	10	7	8	10
TM 2784	37	13	8	8	9	10	2	5
TM 2950	37	13	8	8	9	10	2	5
TM 2960	37	13	8	8	9	10	2	5
TM 2649	50	2	1	4	1	5	2	5
TM 1318	51	2	1	24	33	28	7	22
TM 2072	51	2	1	24	33	28	7	22
SV 373	52	2	8	8	30	26	2	26
SV 445	53	2	8	8	32	26	2	26
TM 2394	54	10	8	8	36	10	25	5
TM 2382	55	11	9	3	10	7	7	10
TM 2494	56	16	1	14	9	10	21	14
LNT 1153	57	16	1	14	9	10	21	15
LNT 1236	57	16	1	14	9	10	21	15
LNT 1189	58	16	9	14	9	10	21	22
LNT 1310	58	16	9	14	9	10	21	22
LNT 1328	58	16	9	14	9	10	21	22
TM 2282	59	18	14	29	19	16	1	6
TM 2868	59	18	14	29	19	16	1	6
LNT 1057	60	25	1	20	18	22	21	21
TM 3192	61	25	1	33	9	7	21	36
LNT 1301	62	26	1	3	6	7	4	15
TM 2914	63	26	1	3	10	7	22	14
LNT 1262	64	26	1	3	18	7	4	22
LNT 1095	65	26	1	3	18	7	22	22
LNT 1201	65	26	1	3	18	7	22	22
TM 2391	66	26	1	8	35	33	24	32
TM 2152	67	26	1	8	39	29	24	30
TM 2532	67	26	1	8	39	29	24	30
TM 2508	68	26	1	14	35	33	21	22
LNT 1240	69	26	1	20	18	23	4	14
TM 3019	69	26	1	20	18	23	4	14
SV 351	70	27	9	21	26	24	2	21
SV 386	71	28	9	25	29	27	2	27
SV 412	71	28	9	25	29	27	2	27
SV 484	71	28	9	25	29	27	2	27
SV 363	72	28	21	22	27	24	2	23
SV 365	73	29	22	23	28	25	2	24
SV 368	74	30	9	24	29	24	2	25
SV 400	75	31	9	26	29	17	23	28
SV 424	75	31	9	26	29	17	23	28
SV 435	76	32	23	27	31	14	2	24
TM 2897	77	36	1	3	35	33	24	32
TM 2289	78	36	1	14	18	31	4	22
TM 2978	78	36	1	14	18	31	4	22
TM 2328	79	37	11	30	28	14	2	17
TM 2415	80	38	1	31	37	34	26	33
TM 2506	81	39	24	32	38	35	14	34
TM 2563	82	40	1	14	40	36	22	14
TM 2223	83	10	8	8	10	10	2	5
TM 2378	84	16	1	14	9	10	24	21
TM 3115	85	16	9	14	9	10	4	21
TM 2549	86	16	9	14	9	10	21	21
TM 2763	86	16	9	14	9	10	21	21
TM 2766	86	16	9	14	9	10	21	21
TM 2767	86	16	9	14	9	10	21	21
TM 2805	86	16	9	14	9	10	21	21
TM 2954	86	16	9	14	9	10	21	21
TM 3106	86	16	9	14	9	10	21	21
TM 3127	87	16	9	14	9	10	22	21
TM 2964	88	16	9	14	9	10	24	21
TM 1307	89	33	1	28	36	1	3	29
TM 2144	90	34	1	3	34	7	24	22
TM 2959	91	41	25	34	41	38	27	35
TM 2965	92	42	26	14	22	37	28	18

### Population structure of *Orientia tsutsugamushi* in Laos

**Fig 2:**
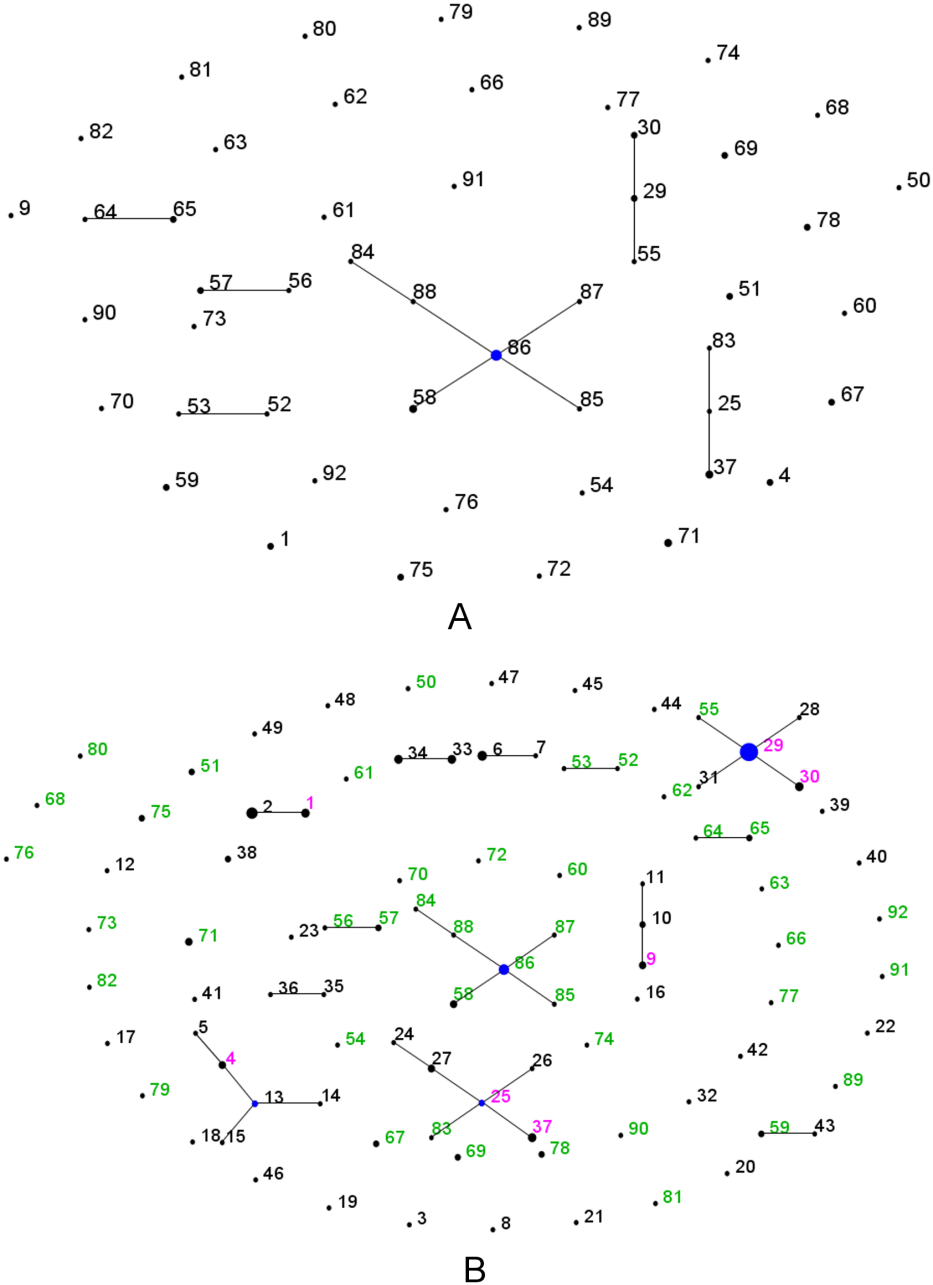
Population genetic structure of *O*. *tsutsugamushi* in Laos and Thailand. (A) *O*. *tsutsugamushi* population in Laos (n = 74). (B) The comparison of *O*. *tsutsugamushi* population in Laos and those in Thailand (n = 89) using comparative e-BURST in which blue dot were founders; pink letter were STs found in both sites; green letter were the STs found only in Laos and black demonstrated the ST found only in Thailand.

**Fig 3:**
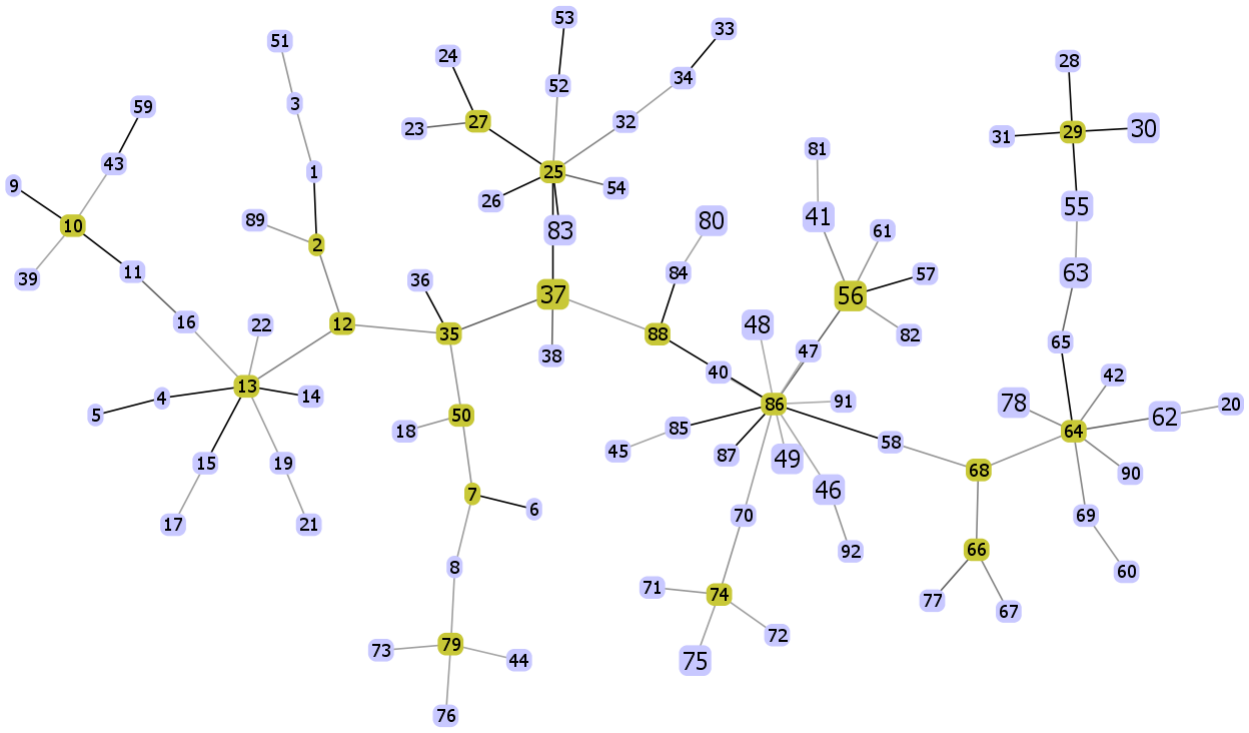
Clonal complex of *O*. *tsutsugamushi* population from Laos and Thailand (163 isolates) using global optimal eBURST (goeBURST) with full minimum spanning tree (MST). ST node colors are followed; green—group founder and light blue—common node.

The MLST data for the 74 isolates from Laos were visualized using eBURST (Fig 2A) and goeBURST (Fig 3). The major difference between these two implementations of the BURST algorithm is that goeBURST provides the option to depict links between STs that differ at more than two loci, whilst eBURST will only show single locus variant (SLV) and double locus variant (DLV) links. Three clonal complexes (CCs) are resolved by eBURST. ST86, which is the most common ST, defines 4 single-locus variants (SLVs) (STs 58, 85, 87, 88) and 1 double locus variant (DLV) (ST84). ST29 is represented by two isolates and also defines two SLVs (ST30, ST55). ST25, which is represented by a single isolate, defines 2 SLVs (ST37, ST83). Three SLV pairs are noted (ST52 and ST53, ST56 and ST57, ST64 and ST65). Relaxing the linkage criteria to double locus variants reveals that the pair ST56 and ST57 are connected to CC86, ST54 is connected to CC25, ST62 and ST63 are connected to the ST64/65 pair, and ST66 and ST77 are joined by a DLV link. Twenty-seven of the 50 STs were not linked to any other STs on the basis of single or double locus variation, which further illustrates the high level of allelic diversity in this population. Analysis using goeBURST also pointed to ST86 as a likely founder (Fig 3), and shows several additional putative links of descent from this genotype by relaxing the criteria for joining STs.

Our data were then compared with previously published data using comparative eBURST (Fig 2B). Besides the seven STs that were noted previously in Thailand and in Vientiane (but not other regions in Laos), there is almost no overlap between the two countries, and only a single SLV link was noted between STs from Thailand (ST43) and Laos (ST59). Although there has been some *O*. *tsutsugamushi* migration between Thailand and Vientiane, as indicated by the 7 shared STs, Vientiane lies across the Mekong river from Udon Thani in Thailand and it is not surprising that STs present in Thailand are also present in patients in this city. However, there is no evidence from this analysis for overlap between the Thai isolates and isolates from elsewhere in Laos.

### Phylogenetic analysis of *Orientia tsutsugamushi* strains in Laos

**Fig 4:**
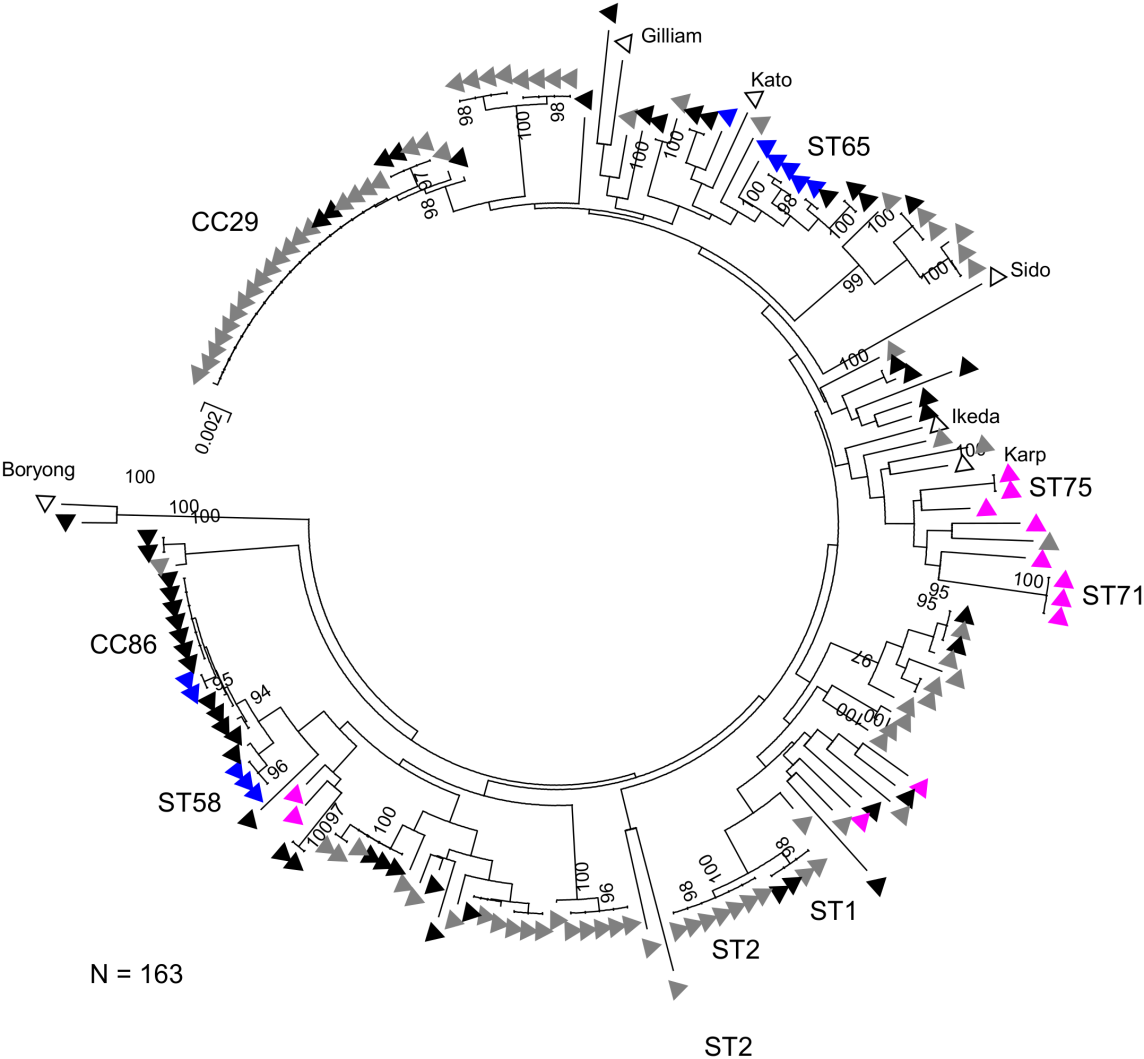
The phylogenetic tree of concatenated sequence of *O*. *tsutsugamushi* isolates from 74 Laos and 89 Thailand using neighbor-joining method with Bootstrap value of 3000. The color triangle demonstrated isolates from different sites: black represented Vientiane; blue represented Luang Namtha; pink represented Salavan; grey were from Thailand and open triangle were reference isolates.

In order to examine further the phylogeny and geographical structuring of the *O*. *tsutsugamushi* isolates, we combined the data for the 74 isolates from Laos with data for 83 strains from Thailand and 6 reference strains (Karp, Kato, Gilliam, Sido, Boryong and Ikeda), giving a total of 163 strains. For each strain, we concatenated the individual allele data, resulting in fragments of 2,700 bp length. This alignment was then used to construct a neighbour-joining tree as implemented in MEGA version 5 (Fig 4).

The clusters resolved by eBURST from both the current and previous Thai study are annotated on the phylogenetic tree. The largest clusters of Thai isolates are CC29 and ST1/ST2, and the tree shows the identical, or very closely related isolates, from Vientiane that correspond to these clusters. The most common clonal complex in the current study, CC86, is confirmed to be composed of a mixture of isolates from Vientiane and Luang Namtha. A second cluster of five isolates from Luang Namtha (including ST65) and three from Vientiane is also noted. In contrast eight of the isolates from Salavan correspond to a single diverse cluster (incorporating ST71 and ST75), which also incorporates a single isolate from Thailand. The other four Salavan isolates are found elsewhere in the tree, but do not correspond to any of the major clusters. The relatively high level of diversity in the major Salavan cluster points to a local population of bacteria circulating in relative isolation in this region over a protracted period of time. In contrast, the more closely related clusters (those of common STs and close relatives that can be identified by eBURST, such as CC29) represent more recent introductions in to a region followed by rapid clonal expansion.

The phylogenetic analysis paints the following general picture regarding geographical structure and spread. Isolates from Vientiane overlap with isolates from Udon Thani and from Luang Namtha, pointing to a key role of the capital both as a focus for movement of isolates between Thailand and Laos and also potentially as a reservoir for spread to and from other parts of Laos. However, there is no evidence for migration between Udon Thani and Luang Namtha. In contrast, the isolates from Salavan appear distinct from all other regions and are less clustered. This indicates that the strains in this region have been diversifying in relative isolation.

**Fig 5:**
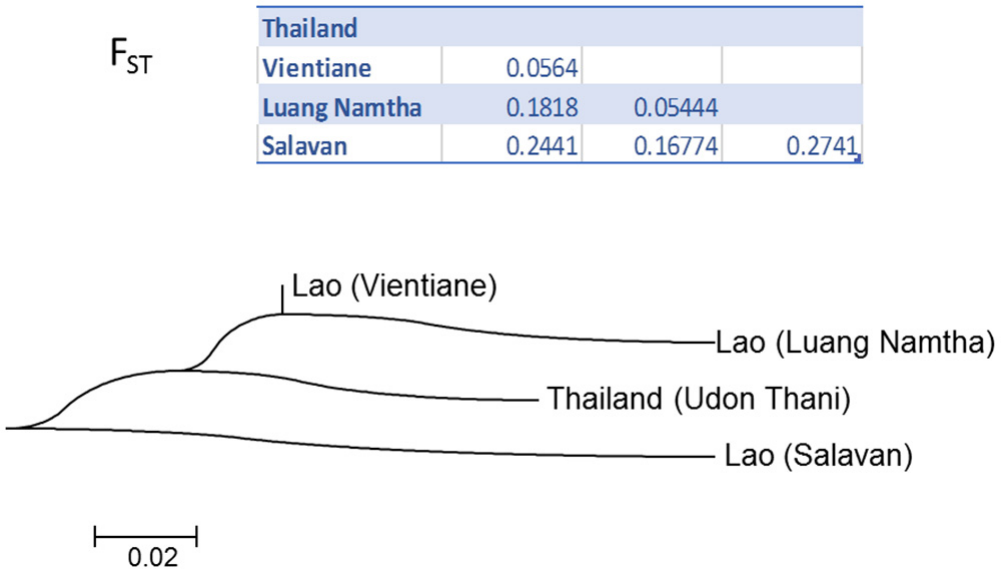
Population differentiation (F_ST_) based on concatenated sequences. Populations were defined according to four strain sources; three from Laos (Vientiane, Luang Namtha and Salavan) characterised in the current study, and one population from Thailand (Udon Thani) characterised previously. The F_ST_ values are depicted as a dendrogram using the neighbour-joining algorithm.

In order to explore this picture further we computed pairwise F_ST_ values for each of the four populations corresponding to Vientiane, Luang Namtha, Salavan and the previously published strains from Udon Thani. These values are given in Fig 5 along with a dendrogram illustrating the level of differentiation between the four populations. Of the 6 pairwise comparisons, two show low levels of differentiation, two moderate and two high. Low levels of genetic differentiation (~0.05) are apparent between the Vientiane and the Thai populations, and between the Vientiane and Luang Namtha populations, consistent with the phylogenetic analysis. A moderate level of differentiation is noted between the Thai and Luang Namtha populations and the Vientiane and Salavan populations (0.17 and 0.18 respectively). Finally, a high level of differentiation is seen between the Salavan population and both the Thai and Luang Namtha populations (0.24 and 0.27 respectively). In summary, this analysis is consistent with the interpretation of the phylogenetic tree in confirming the relative isolation of the Salavan population, and pointing to the Vientiane population as being most “central” (i.e. showing the lowest average differentiation to all other populations).

### Non-synonymous to synonymous nucleotide changes (dN/dS)

The ratios of dN/dS of the 7 loci corresponded to a range of 0.002–0.310 (Table 4), confirming that all genes are evolving predominantly by purifying (stabilizing) selection. This also indicated that synonymous substitutions were more common than non-synonymous substitutions for all the genes tested for these bacteria. We note that the dN/dS ratio of *sucD* and *nrdF* is approximately an order of magnitude lower than the other genes. This was also apparent in the previous data in Thailand [9] and suggests particularly strong selective constraint on these two genes in the *O*. *tsutsugamushi* genome.

**Table 4:** The estimation selection in 7 housekeeping gene using the non-synonymous to synonymous amino acid substitution ratio (dN/dS) in this study.

Locus	Length of allele (bp)	No. of alleles	No. of variable sites	d_N_/d_S_ ^†^
*gpsA*	390	25	42	0.3097
*mdh*	348	13	26	0.206
*nrdF*	384	22	34	0.0021
*nuoF*	360	24	41	0.1111
*ppdK*	396	24	38	0.2316
*sucB*	411	15	28	0.2349
*sucD*	411	25	36	0.0222

^†^ d_N_/d_S_: The ratio of mean non-synonymous substitutions per non-synonymous site and mean synonymous substitutions per synonymous site.

### Recombination

In order to understand how *O*. *tsutsugamushi* was diversified, an estimation of the ratio of recent recombination to mutation events (r/m) in clonal complexes was performed by comparing the sequences of mismatched alleles in clonal founders and single locus variants [23]. The estimated ratio of recombination to mutation of these two populations in Laos (n = 74) and Thailand (n = 89) was high at both the nucleotide level (95:1) and at allele level (17:1), suggesting that the diversification of *O*. *tsutsugamushi* is predominantly characterized by recombination rather than mutation at both nucleotide level and allele level.

## Discussion

This study represents the first investigation into the diversity and phylogeography of *O*. *tsutsugamushi* in Laos. As the isolates were characterized using the same MLST scheme, it was possible to combine these data with those from a previous study focusing on strains from Udon Thani in Northeast Thailand. Our results reveal a highly diverse and recombining population in Laos, as evidenced by the high diversity index (0.98) with high number of STs per isolate (50 STs in 74 isolates, 0.68 STs per isolate). This is consistent with the previous Thai study (0.95 STs per isolate) [9], although the diversity in the current Lao sample set might be expected to be slightly higher, as it represents three distinct geographic sources. The ecological implications of the very high rate of recombination are unclear, but this may reflect co-colonisation of either the mites or the rodents. It is possible that high rates of recombination might also reflect a mechanism for diversification and host adaptation in *O*. *tsutsugamushi* [25]. The *O*. *tsutsugamushi* genome displays a massive proliferation of mobile elements and repeat sequences [26] which are thought to facilitate horizontal gene transfer.

Approximately 8.6% (7/81) of the sequenced isolates appeared to represent mixed infections in patients. This is a lower frequency than in Thailand, where 25% of cultures from patients’ blood were noted to be probable mixed infections. It is possible that the relatively low frequency of mixed infection in the Laos data is an artifact resulting from the *in vitro* culture which may have acted to amplify single predominant strains at the expense of more rare variants over several passages. It is also possible that the predominant isolate was more highly adapted to the culture conditions.

It is not clear whether mixed infection primarily results from multiple mite bites, or from co-colonisation of multiple strains within individual mites. The latter possibility is supported by the detection of multiple antigenic strains of *O*. *tsutsugamushi* in both naturally infected and laboratory-reared chigger mites (*Leptotrombidium* spp.) [27, 28]. As expected, the MLST genes show low dN/dS ratios, which is indicative of stabilizing selection. This is particularly true for *sucD* and *nrdF* which may be under unusual levels of selective constraint. The *sucD* gene produces succinyl-CoA synthase which uses succinyl CoA as a substrate to produce succinate and generate GTP in the citrate pathway (TCA cycle) [29, 30]. Unlike other rickettsia *O*. *tsutsugamushi* has no active pyruvate dehydrogenase enzyme to convert pyruvate to acetyl-CoA (30) and has only a partial TCA cycle starting with *α*-ketoglutarate and ending with oxaloacetate. This ‘minimal’ citric acid cycle requires succinyl-CoA synthase and may explain why *sucD* is relatively conserved in *Orientia*. The ribonucleoside-diphosphate reductase beta subunit gene (*nrdF*) is involved in purine and pyrimidine biosynthesis in *O*. *tsutsugmushi*. The genetic and metabolic diversity in *Rickettsia* has been reported previously [30]. While the proteins unique to *Rickettsia* spp. represent a broad spectrum of functional categories (carbohydrate, lipid transport), more than 60% of the proteins unique to *O*. *tsutsugamushi* belong to the replication, recombination and repair process categories. The *nrdF* gene product is strongly associated with these 3 processes, perhaps explaining why it is relatively conserved in *O*. *tsutsugamushi*.

Limitations of the study include the fact that not all *O*. *tsutsugamushi* isolates underwent MLST and that the patient inclusion criteria for patients recruited in the north and south of the country differed from those recruited in the centre.

Our MLST data reveals a mixed picture concerning geographic structure and migration. First, there is clearly migration and overlap between the strains from Vientiane and from Udon Thani. This is evidenced by shared STs, the intermingling of the isolates on the neighbour-joining tree, and by the F_ST_ analysis. This is perhaps not surprising as these locations straddle the Lao/Thai border. It is possible that disease transmission occurs via the trading activities of villagers in the border area, commuting, or tourism. This may be due to both human movement and movement of mites via animals.

There is no evidence of overlap between the Thai isolates and the Lao isolates from the north (Luang Namtha) or the south (Salavan). There is, however, evidence of transmission between Vientiane and Luang Namtha, particularly with respect to the most common cluster observed in our study, CC86. In contrast, most of the strains from Salavan appear to form a loose cluster which is not closely related to isolates from any of the other regions.

Salavan and Luang Namtha have remained relatively undisturbed rural hinterlands with environments which may be particularly suited to the maintenance of large populations of chigger mites. In contrast, Vientiane (the capital of Laos) is rapidly expanding into surrounding paddy fields and the rural hinterland [31]. This anthropogenic disturbance has likely had a major impact on the life cycle, ecology and behaviour of *O*. *tsutsugamushi* bacteria, their mite vectors, and their rodent and human hosts to limit the spread of the disease. A study on spatial distribution of scrub typhus in Vientiane demonstrated that the prevalence of scrub typhus IgG antibodies among patients from rural villages is significantly higher than that in patients from urban settings. Moreover, many urban patients are positive for *O*. *tsutsugamushi* IgG suggesting prior exposure to scrub typhus, possible in rural settings [31].
