# Correction: The Diversity and Geographical Structure of *Orientia tsutsugamushi* Strains from Scrub Typhus Patients in Laos

**DOI:** 10.1371/journal.pntd.0004742

**Published:** 2016-05-17

**Authors:** Rattanaphone Phetsouvanh, Piengchan Sonthayanon, Sasithon Pukrittayakamee, Daniel H. Paris, Paul N. Newton, Edward J. Feil, Nicholas P. J. Day

One of the affiliations of the first author is missing. Rattanaphone Phetsouvanh is also affiliated with the Department of Clinical Tropical Medicine, Faculty of Tropical Medicine, Mahidol University, Bangkok, 10400 Thailand.
